# Anticancer Activity of the Goat Antimicrobial Peptide ChMAP-28

**DOI:** 10.3389/fphar.2018.01501

**Published:** 2018-12-21

**Authors:** Anna A. Emelianova, Denis V. Kuzmin, Pavel V. Panteleev, Maxim Sorokin, Anton A. Buzdin, Tatiana V. Ovchinnikova

**Affiliations:** ^1^M. M. Shemyakin and Yu. A. Ovchinnikov Institute of Bioorganic Chemistry, The Russian Academy of Sciences, Moscow, Russia; ^2^Department of Bioinformatics and Molecular Networks, OmicsWay Corporation, Walnut, CA, United States; ^3^I.M. Sechenov First Moscow State Medical University, Moscow, Russia

**Keywords:** antimicrobial peptide, cathelicidin, cytotoxicity, cell death, signaling pathways

## Abstract

Cytotoxic effect of the antimicrobial peptide ChMAP-28 from leucocytes of the goat *Capra hircus* was examined against five cancer and two normal human cell lines. ChMAP-28 has the amino acid sequence GRFKRFRKKLKRLWHKVGPFVGPILHY and is homologous to other α-helical mammalian antimicrobial peptides. ChMAP-28 shows considerably higher cytotoxicity against cultured tumor cells than toward normal cells at concentrations of <10 μM. Our findings suggest that ChMAP-28 can initiate necrotic death of cancer cells. Its cytotoxic effect is accomplished due to disruption of the plasma membrane integrity and is not abrogated by the addition of the caspase inhibitor Z-VAD-FMK. ChMAP-28 causes permeabilization of cytoplasmic membrane of human leukemia cells HL-60 already after 15 min of incubation. Here, we show that ChMAP-28 has one of the highest antitumor activity *in vitro* among all known antimicrobial peptides. We speculate that the observed specificity of ChMAP-28 cytotoxic effect against tumor cells is due to its relatively low hydrophobicity and high cationicity. In the meantime, this peptide has low hemolytic activity, which generates a potential for its use as a therapeutic agent.

## Introduction

Cathelicidins are cationic antimicrobial peptides which play a key role in mammalian innate immunity. They were identified first in the mammalian bone marrow myeloid cells. Historically, they were named as “myeloid antimicrobial peptides” (MAPs) (Kościuczuk et al., 2012). The peptides are normally stored as inactive precursors in the secretory granules of neutrophils and macrophages and can be released extracellularly upon leucocyte activation (Zanetti, 2005).

Cathelicidins significantly vary in polypeptide chain length (from 12 to 80 amino acid residues), amino acid sequence, and spatial structure (α-helical or β-sheet, linear or cyclic molecules) (Zanetti, 2005). Among cathelicidins are β-hairpin porcine protegrins with two disulfide bonds and different α-helical peptides (bovine BMAP-27, -28, and -34, ovine SMAP-29 and -34, porcine PMAP-23, -36, and -37, human hCAP18 – LL-37) (Risso et al., 1998, 2002; Kuroda et al., 2015; Lyu et al., 2016). Besides, they may contain a relatively high number of tryptophan residues (indolicidin) (Shagaghi et al., 2016) or prolines (Shamova et al., 1999; Anderson and Yu, 2003). However, all of them have a conserved *N*-terminal part known as the “cathelin” domain, that generally shares of >70% sequence identity to cathelin. The last one was isolated from porcine neutrophils as an inhibitor of cathepsin L (Kopitar et al., 1989). After cleavage of *N*-terminal domain, the peptides exhibit their biological activity (Zanetti, 2005).

Most of them are active against a broad spectrum of microorganisms at micromolar or rather lower concentrations. Recent studies also revealed anticancer activity in some of them (Risso et al., 1998, 2002; Zhang et al., 2015). Their cytotoxic effects are combined and based upon damage of plasma membrane integrity and activation of apoptosis. For example, the bovine α-helical cathelicidins BMAP-27 and BMAP-28 are cytotoxic against human leukemia cells, but also toward proliferating normal human lymphocytes (Risso et al., 1998). BMAP-28 also induced formation of pores in mitochondria and depolarization of its inner membrane (Risso et al., 2002). Addition of BMAP-28 induced apoptosis of human TT thyroid cancer cells and suppressed growth of their xenografts in nude mice (Zhang et al., 2015).

In this study, we investigated a capacity of the cathelicidin from the *Capra hircus* goat leukocytes to induce cell death in tumor and normal mammalian cells. This peptide, designated as ChMAP-28, has been previously predicted bioinformatically based on the sequence data of the precursor protein MAP-28 (GenBank AJ243126.1) (Zhang et al., 2014), but has never been purified and/or investigated experimentally as an individual peptide. The ChMAP-28 amino acid sequence was translated from mRNA for the corresponding precursor protein as a 27-residue peptide without the *C*-terminal glycine, a common amidation signal in cathelicidins. It has molecular mass of 3365 Da and the amino acid sequence GRFKRFRKKLKRLWHKVGPFVGPILHY including eleven basic residues. The goat peptide is highly homologous to the bovine α-helical cathelicidin BMAP-27. We expressed the recombinant ChMAP-28 in *Escherichia coli* and examined its cytotoxic properties against four mammalian cancer cell lines (HL-60 acute promyelocytic leukemia, A431 human epidermoid carcinoma, B16F1 murine melanoma, SKBR-3 human breast adenocarcinoma), one transformed human cell line (HEK 293T transformed human embryonic kidney) and two types of primary cultures of normal human cells (HEF human embryonic fibroblasts and NHA normal human astrocytes). We investigated its specificity and the mechanism of cytotoxicity in the populations of susceptible cells. ChMAP-28 was found to be toxic toward tumor cells at concentrations of <10 μM, significantly less toxic toward normal cells and low hemolytic. The cytotoxic effect of ChMAP-28 is revealed in 15 min and does not increase during following incubation. We showed that its cell death promoting mechanism was not associated with the caspase-dependent apoptosis, but was rather related to necrosis due to pore formation in the susceptible cell membranes.

## Materials and Methods

### Cell Lines and Culture Conditions

The following mammalian cell lines were used in this study: HL-60 (acute promyelocytic leukemia), A431 (human epidermoid carcinoma cells), B16F1 (murine melanoma), SKBR-3 (human breast adenocarcinoma cells), HEK 293T (transformed human embryonic kidney cells), HEF (human embryonic fibroblasts), and NHA (normal human astrocytes). All cancer and transformed cell lines were obtained from the American Type Culture Collection (ATCC)^1^. Primary cells HEF (normal human embryonic fibroblasts derived from human embryonic stem cells) and NHA (normal human astrocytes derived from human fetal brain tissue) were obtained and cultured according to the corresponding protocols (Xu et al., 2004; Xiong and Gendelman, 2013). Peripheral blood mononuclear cells (PBMC) were isolated from fresh human whole peripheral blood according to the protocol (Dagur and McCoy, 2015) and used for the cytotoxicity experiments immediately after isolation. Briefly, cells were cultured in DMEM/F12 (1:1) or RPMI-1640 medium containing 10% fetal bovine serum (FBS) (“Invitrogen”) at 37°C in the atmosphere containing 5% CO_2_ and 95% air according to standard mammalian tissue culture protocols and using sterile technique. All cell lines were tested before use by LookOut^®^ *Mycoplasma* PCR Detection Kit (“Sigma-Aldrich”) according to the manufacturer’s protocol and found to be free of *Mycoplasma* infection.

### Peptides

The peptide primary structure was deduced from the mRNA sequence of the corresponding precursor protein (GenBank: AJ243126.1). Oligonucleotides were designed on the basis of *E. coli* codon usage bias. *E. coli* BL21 (DE3) cells transformed with the constructed plasmid were grown up to OD_600_ 1.0 and then were induced with 0.2 mM IPTG. The induction was performed at 30°C for 5 h under stirring with a shaking speed of 220 rpm. The peptide purification included immobilized metal affinity chromatography (IMAC) of cell lysate, CNBr cleavage of the fusion protein, and reversed-phase HPLC as described previously (Panteleev et al., 2015). The reversed-phase HPLC fractions were dried *in vacuo*, dissolved in water and analyzed by MALDI-MS (Bruker Daltonics). The obtained non-amidated recombinant analog of natural ChMAP-28 was analyzed by automated microsequencing with the use of the Procise cLC 491 Protein Sequencing System (PE Applied Biosystems) (data not shown). Melittin (>98% pure) was synthesized using a standard solid-phase method and provided by Dr. Sergey V. Sychev. The peptides concentrations were estimated on the basis of near-UV absorbance measurement and calculated with the use of extinction coefficients.

### Hemolytic Activity Assay

Hemolytic activity of ChMAP-28 and melittin was tested against fresh human red blood cells (hRBC) washed three times with phosphate buffered saline (PBS: 10 mM Na_2_HPO_4_, 1.76 mM K_2_HPO_4_, pH 7.4, containing 173 mM NaCl, and 2.7 mM KCl). Two-fold serial dilutions of the peptides solutions were then added to 50 μl aliquots of hRBC in PBS to adjust a final volume to 100 μl and hRBC concentration to 4% (v/v) in each well of a 96-well plate. The suspension was incubated for 1.5 h at 37°C under stirring at 1000 rpm. The plates were centrifuged at 2000 *g* for 5 min. Supernatant aliquots of 50 μl were transferred into flat-bottomed 96-well microplates, and the release of hemoglobin was monitored by measuring the absorbance at 405 nm in a microplate reader (Eppendorf, Germany). hRBC in PBS and 0.1% Triton X-100 were used as negative and positive controls, respectively. Hemolytic activity was expressed as a percentage of hemolysis calculated according to the following equation:

$$\mathrm{Hemolysis}\;(\%)=({\mathrm{OD}}_{405}\;\mathrm{sample}-{\mathrm{OD}}_{405}0\%\;\mathrm{lysis}\;\mathrm{control})/({\mathrm{OD}}_{405}\;100\%\;\mathrm{lysis}\;\mathrm{control}-{\mathrm{OD}}_{405}\;0\%\;\mathrm{lysis}\;\mathrm{control})\times 100\%$$

Two experiments were performed with fresh hRBC from human blood samples of independent donors. The quantitative data were represented as average means with standard deviations.

### Cytotoxic Activity Assay

The colorimetric 3-(4,5-dimethylthiazol-2-yl)-2,5-dipheny-ltetrazolium bromide (MTT) dye reduction assay was used to determine the cytotoxicity of the ChMAP-28. 5 × 10^3^ – 10^4^ cells per well for the cell lines and 10^5^ cells per well for PBMC in DMEM/F12 or RPMI-1640 medium, supplemented with 10% FBS were placed into 96-well plates and then incubated in the atmosphere containing 5% CO_2_ and 95% air at 37°C overnight. After the media were removed, ChMAP-28 was dissolved in 0.1 ml DMEM/F12 with 10% FBS. The peptide was added to the cell cultures up to final concentrations of 0.6, 1.25, 2.5, 5, 10 μM. Melittin was added at final concentrations of 0.15, 0.3, 0.6, 1.25, 2.5, 5 μM. HL-60 cells (4 × 10^4^ in 50 μl) were added to two-time serial dilutions of ChMAP-28 or melittin solutions in 50 μl RPMI-1640 with 10% FBS. Forty eight hours later, 20 μl of MTT (5 mg/ml) was added into each well and the plates were incubated for 3 h at 37°C. The plates with HL-60 cells were centrifuged for 10 min at 2000 rpm. The media were discarded and 0.1 ml of dimethyl sulfoxide and isopropanol mixture at a ratio of 1:1 (v/v) was added to each well to dissolve the crystallized formazan. The absorbance at 570 nm was measured by a microplate reader (Eppendorf).

### Annexin V-FITC/Propidium Iodide Double Staining and Flow Cytometry

Cell death analysis with annexin V-FITC/propidium iodide (PI) double staining and dead cells counting with flow cytometry were performed 1, 2, and 4 h after addition of ChMAP-28 up to a final concentration of 1, 3, or 6 μM or mellitin up to a final concentration of 1, 2, or 4 μM. The Annexin V FITC Apoptosis Detection Kit (BD Biosciences, United States) was used according to the manufacturer’s protocol on NovoCyte flow cytometer (ACEA Biosciences, United States). Each experiment was performed in triplicate. The apoptosis inducer camptothecin (Sigma-Aldrich) and general caspase inhibitor Z-VAD-FMK (BD Biosciences, United States) were used. All flow cytometry experiments were performed twice.

### Trypan Blue Exclusion Assay

ChMAP-28 or melittin were diluted in non-supplemented RPMI medium and incubated with 4 × 10^5^ HL-60 cells in a volume of 100 μl. After 15 min or 1 h of incubation with 3 or 6 μM ChMAP-28 or 2 or 4 μM melittin, proportions of viable cells were counted in a hematocytometer using sterile-filtered 0.4% trypan blue solution. The quantitative data were represented as average means with standard deviations (±SD) obtained from three independent experiments.

### Lactate Dehydrogenase (LDH)-Release Assay

The LDH-release assay (CytoTox 96 Non-Radioactive Cytotoxicity Assay; Promega Corporation, United States) was performed according to the manufacturer’s protocol. Suspension HL-60 cells (4 × 10^4^) were seeded into a 96-well plate in triplicate and incubated for 1 h at various concentrations of ChMAP-28 or melittin at 100 μl per well in the RPMI serum-free medium. Absorbance was measured at 492 nm using a microplate reader (Eppendorf, Germany). Percentage of LDH-release was calculated according to the following formula:

[(experimental LDH−release−spontaneous LDH​release)/(maximal LDH−release−spontaneous LDH−release)]×100%.

Dose-response curves were plotted as an average of three independent experiments.

### Statistical Analysis of Cell Culture Data

Statistical analysis was performed using the GraphPad PRISM 6.0 software (GraphPad Software Inc.), and values of *p* < 0.05 were considered statistically significant. The data were represented as the mean ± SD of three independent experiments.

### Synthesis of Microarrays

B3 microarray synthesizer (CustomArray, United States) was used for forty nucleotides-long oligonucleotide probe synthesis on CustomArray ECD 4X2K/12K slides. Synthesis was performed according to the manufacturer’s recommendations. Two replicates of total 6020 unique oligonucleotide probes specific to 3706 human gene transcripts were placed on each chip. Chip design was performed using the Layout Designer software (CustomArray, United States). For the custom microchip, we used original oligonucleotide probe sequences of the Illumina HT 12 v4 platform.

### Library Preparation and Hybridization

The complete Whole Transcriptome Amplification WTA2 kit (Sigma) was used for reverse transcription and library amplification. The manufacturer’s protocol was modified by adding to amplification reaction of dNTP mix containing biotinylated dUTP, resulting to a final proportion of dTTP to biotin-dUTP as 5/1. Microarray hybridization was performed according to the CustomArray ElectraSense^TM^ Hybridization and Detection protocol. Hybridization mix contained 2.5 μg of the labeled DNA library, 6X SSPE, 0.05% Tween-20, 20 mM EDTA, 5× Denhardt solution, 100 ng/μl sonicated calf thymus gDNA, 0.05% SDS. The hybridization mix was incubated with a chip overnight at 50°C. Hybridization efficiency was detected electrochemically using the CustomArray ElectraSense^TM^ Detection kit and ElectraSense^TM^ 4X2K/12K Reader.

### Initial Processing of Microarray Data

Probe signals were geometrically averaged, thus obtaining expression value for each specific type of the probe. Then quantile normalization (Bolstad et al., 2003) was performed using the “preprocessCore” R package (Bolstad, 2018).

### Functional Annotation of Gene Expression

The Oncobox knowledge base was used to determine structures of intracellular molecular pathways as described previously (Spirin et al., 2014). We applied the original Oncobox algorithm (Buzdin et al., 2014, 2017) for functional annotation of the primary expression data and for calculating pathway activation strength (PAS) scores and cancer-to-normal ratios (CNRs). CNRn is the ratio of the expression levels of a gene n in the sample under investigation to the average expression in the control group of samples. In this study, the PAS scores were obtained according to Buzdin et al. (2014). PAS can take both positive and negative values meaning over- or underactivation relative to controls.

## Results

### Expression and Purification of the Recombinant ChMAP-28

To improve a yield of the recombinant peptide in *E. coli* and to facilitate its purification, ChMAP-28 was obtained as a part of the fusion protein with the *N*-terminal octahistidine tag and the modified thioredoxin A (M37L). Following cell harvesting, sonication, preparative centrifugation of the cell lysate, affinity chromatography and specific CNBr cleavage of the fusion protein, the target peptide was purified by reversed-phase HPLC in a linear gradient of acetonitrile concentration. MALDI mass spectrometry analysis of the main fraction showed that the measured monoisotopic m/z (3364.2) matched well the calculated molecular mass of the protonated ion [M+H]^+^ of the ChMAP-28 (3364.0 Da) (Supplementary Figure S1). The final yield of ChMAP-28 was ∼3.1 mg per 1 l of bacterial culture.

### Hemolytic Activity of ChMAP-28

**FIGURE 1:**
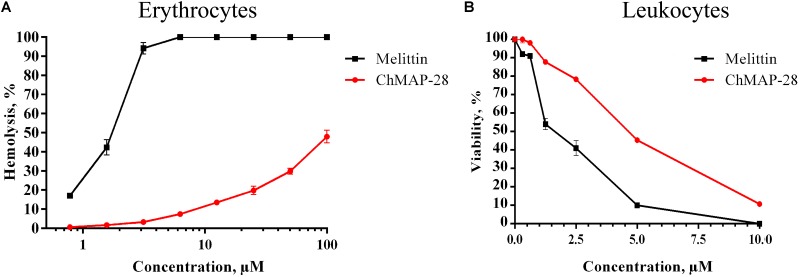
Toxicity of ChMAP-28 and melittin to normal human blood cells. **(A)** Concentration-dependent curve of ChMAP-28 and melittin toward PBMC assessed by MTT-test. **(B)** The hemolysis curve showing effects of ChMAP-28 and mellitin on human erythrocytes. The data represent the mean values ± SD of three independent series of triplicated experiments.

The assay for hemolytic activity of ChMAP-28 was performed to determine the peptide effect on the integrity of red blood cells. This assay allows to assess an ability of the peptide to permeabilize cytoplasmic membrane of erythrocytes. Their lysis results in a detectable increase of the optical density caused by hemoglobin release into solution. For both ChMAP-28 and melittin, we used the detergent Triton X-100 as a positive control and the PBS buffer solution as a negative control. The results (Figure 1A) evidence that ChMAP-28 has a low hemolytic activity (hemolysis less than 10%) at a concentration range up to 10 μM. The half-hemolysis concentration of ChMAP-28 determined in this study (∼100 μM) was ∼20-fold higher than its IC_50_ measured against the cancer cell lines and shown in Table 1. In contrast, melittin caused 42% hemolysis at the peptide concentrations down to 1.5 μM.

**Table 1:** Comparison of IC_50_ values determined for ChMAP-28 and melittin.

Cell line	HL-60	SKBR-3	A431	B16F1	HEK293T	HEF	NHA
	
Peptide	IC_50_, μM						
ChMAP-28	3.39 ± 0.15	5.63 ± 1.05	6.49 ± 0.09	4.82 ± 1.01	5.09 ± 0.40	8.95 ± 2.68	ND (>10)
Melittin	1.86 ± 0.03	2.03 ±0.09	1.09 ± 0.17	1.46 ± 0.07	1.42 ± 0.18	1.64 ± 0.10	1.83 ± 0.15


*IC_*50*_ values are represented as the means ± standard deviations (SD) of at least three independent experiments. In the case of NHA cells, 50% inhibition was not reached even at the peptide concentration of 10 μM, thus the IC_*50*_ value could not be determined.*

### Cytotoxicity of ChMAP-28

**FIGURE 2:**
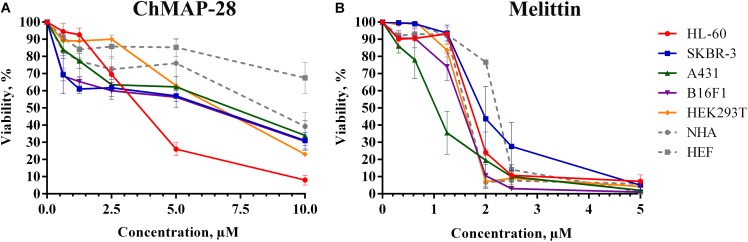
The concentration-dependent cytotoxicity curves of ChMAP-28 **(A)** and melittin **(B)** toward cancer and normal cell lines assessed by the MTT-test. The data are presented as the mean ± standard deviation (SD) of at least three independent experiments.

We evaluated the cytotoxic activity of ChMAP-28 against seven mammalian cell types. Four cancer (HL-60 acute promyelocytic leukemia, A431 human epidermoid carcinoma, B16F1 murine melanoma, SKBR-3 human breast adenocarcinoma), one transformed (HEK 293T transformed human embryonic kidney) and two normal human cell lines (HEF human embryonic fibroblasts and NHA normal human astrocytes) were used in this study. HEF and NHA cells were chosen as *in vitro* normal cell models. Cytotoxicity was measured by incubating the cells with serial dilutions of ChMAP-28 followed by MTT assay after 48 h. Dose-dependent cytotoxicity effect curves for the cell lines investigated are shown in Figure 2A. As the positive control, we used the α-helical antimicrobial peptide melittin with a pronounced lytic activity (Figure 2B). For every condition, cell viabilities were assessed in at least three independent experiments for every cell culture. The half maximal inhibitory concentrations (IC_50_) were measured as the peptide concentration at which cell viability was reduced by 50% in comparison to untreated cells (Table 1). These results demonstrate that ChMAP-28 has a pronounced cytotoxic activity against the mammalian cancer and transformed cell lines and a significantly reduced effect on the normal cells. The most significant cytotoxicity was observed for the HL-60 leukemia cells (IC_50_ = 3.4 μM). In contrast, the normal cells (HEF fibroblasts and NHA astrocytes) were much less sensitive to ChMAP-28, with IC_50_ of ∼9 μM for HEF, and of >10 μM for NHA cells. In contrast, the control cytolytic peptide melittin displayed cytotoxicity against all cell types revealing IC_50_ within a narrow range of 1–2 μM without any selectivity toward adherent, suspension, cancer or normal cells. Toxicities of ChMAP-28 and mellitin measured by MTT assay toward freshly isolated human PBMC were compared in Figure 1B. The IC_50_ value for ChMAP-28 (4.6 μM) was lower than the half-hemolysis concentration HC_50_ value (∼100 μM), but was still higher than that measured toward tumor suspension cells HL-60 (3.4 μM). At the same time, the melittin cytotoxicity was almost equal for PBMC and HL-60 cells (IC_50_ values for both cell types were of 1.9 μM and HC_50_ value was of 1.6 μM).

### Mechanism of ChMAP-28 Cytotoxicity

According to MTT-test, HL-60 cells were found to be the most sensitive to ChMAP-28. They were used, therefore, for investigation of cell death mechanisms with the use of flow cytometry.

FITC-Annexin V binds to phosphatidylserine, which is exposed on the outer leaflet of the cellular plasma membrane at the initial stages of apoptosis, whereas PI preferentially stains nuclei of dead cells. Therefore, a combination of FITC-Annexin V and PI assays can be conclusive for differentiating between early apoptotic and late apoptotic/necrotic cells.

ChMAP-28 was used at three concentrations: less than IC_50_ (1 μM), comparable to IC_50_ (3 μM) and twofold exceeding IC_50_ (6 μM). Melittin was used as the positive cytotoxic control at final concentrations of 1, 2, and 4 μM. Three time points were used for measurements of the peptide-dependent cytotoxicities: 1, 2, and 4 h after addition of the peptides.

**FIGURE 3:**
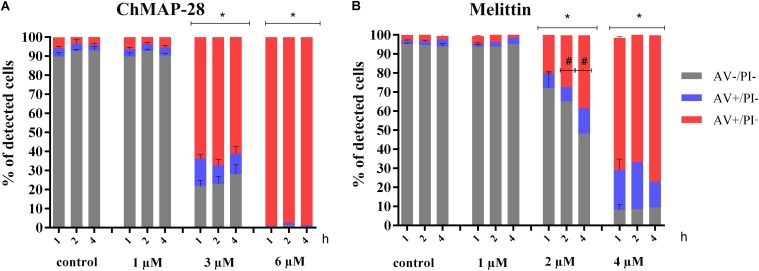
The flow cytometry results of FITC-annexin V/PI double staining of HL-60 cells after **(A)** 1, 2, and 4 h of incubation with RPMI-1640 medium (control) at the final ChMAP-28 concentrations of 1, 3, and 6 μM. **(B)** 1, 2, and 4 h of incubation with RPMI-1640 medium (control) at the final melittin concentrations of 1, 2, and 4 μM. The results are presented as the percentage of viable (AV- PI-), apoptotic (AV+ PI-), secondary apoptotic/necrotic (AV+PI+) cells. (^∗^*p* < 0.05 vs. the control samples, *^#^p <* 0.05 for AV- PI- and AV+PI+cells vs. 1 h samples, respectively).

The results of double staining of HL-60 cells, followed by the flow cytometry analysis are shown in Figure 3. The ChMAP-28 concentration of 1 μM was not toxic to HL-60 cells. However, their incubation with ChMAP-28 at the peptide concentration of 3 μM for 1, 2, and 4 h caused disruption of membrane integrity in >60% of the cells. Moreover, at the ChMAP-28 final concentration of 6 μM almost all cancer cells were dead at any time points. Thus, the effect of ChMAP-28 on cancer cells depended mostly on the peptide concentration but not on the incubation time (Figure 3A) which was illustrative of the peptide nondelayed effect.

Melittin was not cytotoxic at the concentration of 1 μM but, in contrast, at the peptide concentration of 2 μM the percentage of dead cells increased exponentially with the incubation time (Figure 3B).

To further investigate the effect of ChMAP-28, we performed double staining assay also for the adhesive cell line HEK293T. After incubation for 4 h with ChMAP-28 at about the twofold IC_50_ concentration (10 μM), >70% of the HEK293T cells underwent the late stages of apoptosis/necrosis (data not shown).

### Inhibition of Caspases

**FIGURE 4:**
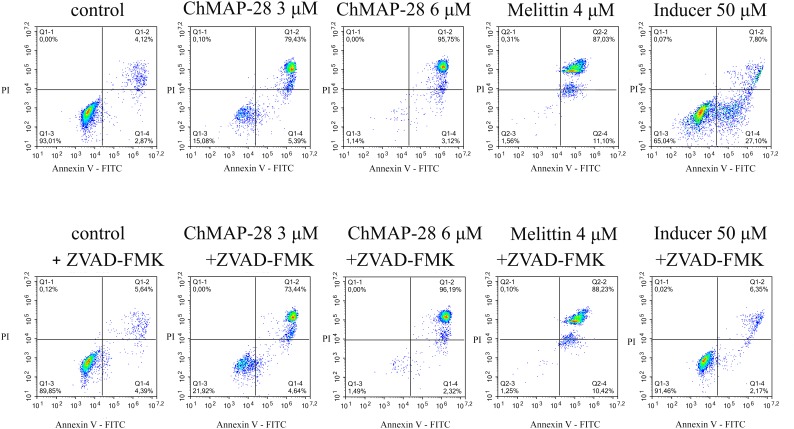
Cytotoxicity of ChMAP-28 in the presence of the caspase inhibitor Z-VAD-FMK. Columns from left to right: control; 3 μM ChMAP-28; 6 μM ChMAP-28; 4 μM melittin; 50 μM apoptotic inducer campthotecin. Top row: no Z-VAD-FMK was added, bottom row: 50 μM Z-VAD-FMK was added.

In order to elucidate the mechanism of action of ChMAP-28, we applied a widely used caspase inhibitor Z-VAD-FMK in the flow cytometry assay with FITC-Annexin V/PI staining. ChMAP-28 was used at two final concentrations: 3 and 6 μM, cytotoxic control melittin was used at a final concentration of 4 μM. The membrane-permeabilizing capacity of ChMAP-28 was not affected in the presence of Z-VAD-FMK after 4 h of incubation (Figure 4). With that, more than 70% of dead double-stained cells at the ChMAP-28 concentration of 3 μM and 96 % at the peptide concentration of 6 μM were detected. Melittin also showed similar effects both in the presence or in the absence of Z-VAD-FMK. In contrast, Z-VAD-FMK totally abrogated the effect of 50 μM apoptotic inducer campthotecin, used as a positive control.

### Trypan Blue Assay for Dead Cells

**FIGURE 5:**
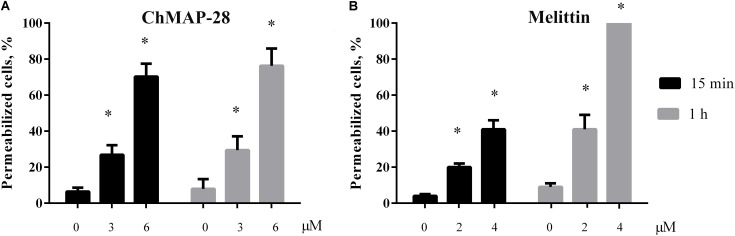
Trypan blue exclusion assay of HL-60 cell death after 15 min (black bars) or 1 h (gray bars) of incubation with ChMAP-28 **(A)** and melittin **(B)**. (^∗^*p* < 0.05 vs. the control sample 0 μM for each time interval, respectively).

We assessed a short-term membrane permeabilizing effect of ChMAP-28 on HL-60 cells using the trypan blue exclusion assay. The experiments were done after 15 min and 1 h of incubation with ChMAP-28. Previously, the proportion of dead HL-60 cells was virtually unchanged after 1, 2, and 4 h of incubation in the flow cytometry experiments. In the control experiments (cells without addition of ChMAP-28), cell death did not exceed 10%. At the ChMAP-28 final concentration of 3 μM 27 and 28% of trypan-stained (dead) cells were detected after 15 min and 1 h incubation, respectively. For 6 μM ChMAP-28, this proportion amounted to 71 and 76%, respectively. We concluded, therefore, that the ChMAP-28-induced cell death occurs mostly during the first 15 min (Figure 5A), which is typical of necrotic cell death (Janko et al., 2013). In contrast, for melittin the proportion of dead cells was changed in both time- and concentration-dependent manners (Figure 5B). Taken together with the previous data, these findings evidence that ChMAP-28 induces necrotic, but not apoptotic death of HL-60 cells.

### LDH-Release Assay for Measurement of Cell Permeability

**FIGURE 6:**
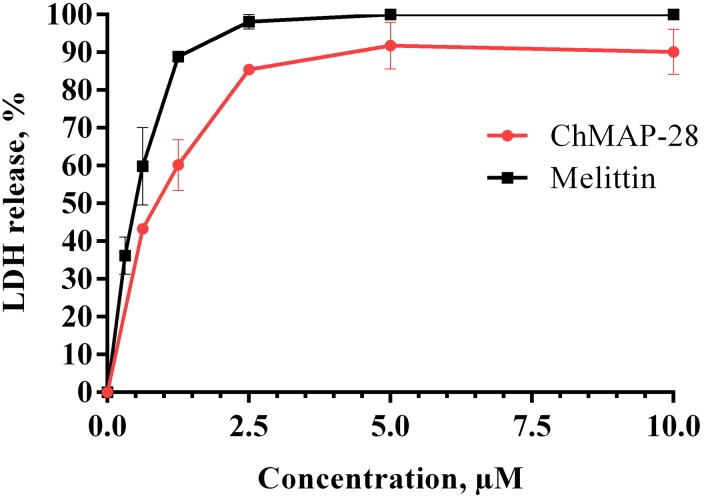
Lactate dehydrogenase assay of HL-60 cells treated with ChMAP-28 or melittin. Data represent the mean values ± SD for three independent series of triplicated experiments.

Membrane permeability evaluated with trypan blue exclusion assay and annexin V-FITC/PI double staining was also measured by lactate dehydrogenase (LDH)-release (Chan et al., 2013; Maes et al., 2015). Cytosolic enzyme LDH is released upon cell lysis and therefore can be a marker of cell integrity. Percentage of LDH-leakage from cells enables to evaluate a direct cell lysis. The obtained results showed that ChMAP-28 and melittin induced a concentration-dependent lysis in HL-60 cells within 1 h of incubation (Figure 6). We found that both agents caused comparable levels of LDH-release, with slightly higher levels for melittin (Figure 6).

### Analysis of Molecular Pathways

To clarify the molecular mechanisms of ChMAP-28 cytotoxicity, we profiled gene expression in HL-60 cells, both treated with ChMAP-28 and unaffected by the peptide. Each measurement was done in triplicate at the ChMAP-28 final concentrations of 1.25, 2.5, and 5 μM. To this end, the total RNA preps were extracted, and gene expression was screened using microarray hybridization. We used original customized microchip system developed on the basis of the CustomArray (United States) technology of direct electrochemical oligonucleotide synthesis in the array (Lodes et al., 2009). Using the CustomArray platform, we synthesized the arrays with 2228 oligonucleotide probes corresponding to 2016 human genes involved in 334 intracellular signaling pathways (Supplementary Table S1). For the custom microchip, we used original oligonucleotide probe sequences of the Illumina HT 12 v4 platform. The microarray hybridization signals were quantile normalized according to Bolstad et al. (2003) and gene expression data were deposited in GEO database with the accession number GSE118397.

Then, we processed the gene expression data to establish normalized case-to-normal (CNR) expression rates for each gene and to build PAS profiles corresponding to intracellular signaling pathways for each sample. The analysis included 334 pathways (Supplementary Table S1) and 2016 individual gene products. For PAS measurements, we applied the Oncobox method which was previously shown to increase the stability of gene expression data (Borisov et al., 2017) and to be applicable to the analysis of various types of biosamples (Buzdin et al., 2017). The formula for PAS calculation for a given pathway (*p*) is as follows: *PAS*_p_ = (∑_n_ *ARR*_np_ ⋅ lg(*CNR*_n_))/N (Buzdin et al., 2014). The functional role of a gene product in a pathway is reflected here by a discrete flag *activator/repressor role* (*ARR*), which equals 1 for an activator, –1 for a repressor, and shows intermediate values of -0.5; 0.5; and 0 for the gene products having intermediate repressor, activator, or unknown roles, respectively. The *CNR_n_* value is the ratio of the expression level of a gene *n* in the sample under investigation to the average expression level in the control sampling. N is the number of individual gene products in the pathway p. The positive value of PAS indicates activation of a pathway, and the negative value – its repression in a biosample under investigation. The CNR and PAS values obtained for the normal and peptide-treated samples are shown in Supplementary Table S1.

In accordance with the above-stated data, we did not detect activation of apoptotic pathways in the ChMAP-28-treated cells. However, we detected a number of molecular pathways most likely implicated in the survival of ChMAP-28-treated cells. For example, increasing concentrations of the peptide gradually upregulated activity of AKT signaling pathway. Several other pathways were also upregulated at higher concentrations of ChMAP-28: a branch of Wnt signaling pathway promoting cell survival and ILK (Integrin-linked kinase) pathway. On the other hand, the GSK3 (Glycogen synthase kinase 3) pathway, Notch and PTEN signaling pathways were downregulated while ChMAP-28 was added. PAK (p21-activated kinases) signaling was slightly decreased at moderate concentrations of ChMAP-28, but strongly upregulated at high concentrations of >IC_50_. Their activation patterns may reflect the molecular mechanisms recruited by HL-60 cells to survive in the presence of the ChMAP-28 molecules. Of note, most of these pathways deal with the interplay of cytoskeleton and membrane proteins, which is in line with the cytoplasmic membrane permeabilization mechanism by ChMAP-28.

In complete agreement with a well-known positive connection of PTEN and apoptosis (Yao et al., 2018), downregulation of PTEN pathway provided an additional line of evidence for a non-apoptotic mechanisms of ChMAP-28 cytotoxicity.

## Discussion

In this study, for the first time we characterized cytotoxic activity of cathelicidin antimicrobial peptide ChMAP-28. Here, we expressed the recombinant ChMAP-28 peptide and investigated its activity toward mammalian cancer and normal cells. Our data evidence that this natural innate immunity effector molecule ChMAP-28 has significant cytotoxic activity, manifested primarily against tumor cells. The most pronounced effect was observed against human leukemia cell line HL-60. The apparent selectivity of ChMAP-28 toward cancer and transformed vs. normal cells wfas further confirmed by the significantly lower toxic effect toward fresh human red blood cells. The effect was investigated in comparison with that of melittin – the main peptide component of honeybee venom.

Based on timing parameters of the observed cytotoxicity of ChMAP-28, we showed that the cytotoxic effect may develop in a short period of time – less than in 15 min after addition of the peptide to the culture medium. Further, we showed that the ChMAP-28 cytotoxic effect is most likely mediated by the necrotic rather than apoptotic mechanism, due to the fast cellular cytoplasmic membrane permeabilization by ChMAP-28.

Recently, it has been shown that tumor cells are typically deficient in glycosylation of their extracellular membrane-bound proteins and aggregates as compared with normal cells (Alam et al., 2017). This leads to a greater accessibility of membranes and their components to foreign proteins, e.g., antibodies (Pochechueva et al., 2016). Relaxed glycosylation profile can be, therefore, also a reason for a greater accessibility of tumor cells to ChMAP-28, in particular, for its tumor-specific cytotoxic pattern.

ChMAP-28 is the first cathelicidin with a pronounced antitumor activity derived from goat leukocytes. Other caprine cathelicidins, e.g., proline-rich peptides bactenecins and mini-bactenecins from *Capra hircus* neutrophils showed lower cytotoxicity. ChBac3.4 and ChBac5 demonstrated very low hemolytic activity, even at relatively the concentrations of up to 100 μM. However, ChBac3.4 showed selective cytotoxicity toward two mammalian suspension cell cultures K562 and U937, but had a low toxicity against adherent normal and tumor cells – human lung carcinoma A549, human embryonic lung fibroblasts MRC-5 and normal human skin fibroblasts in broad concentration ranges (Shamova et al., 2009).

Similar to ChMAP-28, the shortened caprine cathelicidine peptides – proline-rich minibactenecins mini-ChBac7.5Nα and mini-ChBac7.5Nβ did not show lytic effect on human red blood cells and were non-toxic to various cultured mammalian cells (Shamova et al., 2016). ChMAP-28 also has advantages over β-hairpin antimicrobial peptides. First, ChMAP-28 is active in a lower concentration range (of <10 μM), while β-hairpin peptides are effective at concentrations of 20–100 μM (Kuzmin et al., 2018). Another disadvantage of β-hairpin antimicrobial peptides is the high hemolytic activity accompanying their antitumor effects.

Among all cathelicidines only the bovine peptide BMAP-28 which is highly homologous to ChMAP-28 has a comparable cytotoxic activity at relatively low concentrations (IC_50_ of 1.5–6 μM against suspension cells K562). However, BMAP-28 is significantly more hemolytic (its HC_50_ is of ∼15 μM, vs. ∼100 μM for ChMAP-28) (Risso et al., 1998; Ahmad et al., 2009). BMAP-28 is also more toxic for normal mammalian cells (its IC_50_ is less than 4 μM after incubation with murine fibroblasts (Ahmad et al., 2009), vs. >8 μM for ChMAP-28). The best known human α-helical cathelicidin LL-37 has a lower cytotoxic activity toward both tumor and normal cells (IC_50_ of >10 μM and HC_50_ of ∼60 μM) (Oren et al., 1999). IC_50_ of LL-37 against colon carcinoma cells were in a range of 20–60 μM (Ren et al., 2012, p. 37). Thus, ChMAP-28 seems to be the best representative of the cathelicidins so far in terms of specific antitumor toxicity.

The mechanism of the ChMAP-28 action does not comprise apoptosis of the susceptible cells, as evidenced by high throughput profiling of gene expression during cell exposure to this peptide. There were no major pro-apoptotic signaling pathways activated by ChMAP-28, in contrast to multiple pro-survival pathways that were upregulated in these cells, such as p38 and AKT-signaling pathways (Supplementary Table S1). This most probably reflects corrective action to overcome ChMAP-28 toxic effects and retain viable status of the cells. However, exposure to ChMAP-28 resulted in downregulation of GSK3 and NOTCH3 receptor pathways, which may suggest depressed metabolism and cell contacts under stressful conditions (Supplementary Figure S2).

Pathway analysis revealed inactivation of entire Caspase Cascade Pathway (Supplementary Figure S3), which means that expression level of most genes involved in this pathway is less than in control samples. In addition, we observed the sequential ChMAP-28-dependent increase of PARP1 expression (Supplementary Table S1. Sheet “CNR”), which mediates necrosis (Moubarak et al., 2007; Shin et al., 2015). Moreover, we did not observe an increase of expression for p53 targets, such as TP53AIP1, PMAIP1. Expression level of these genes was below normal levels in most cases (Supplementary Table S1. Sheet “CNR”). Thus, we concluded that there was no p53 activation during ChMAP-28 administration. In addition, we observed decreased levels of CASP6 and CASP10. Expression levels of these caspases were previously shown to be linked with apoptosis (MacLachlan and El-Deiry, 2002; Rikhof et al., 2003; Kida et al., 2006). Together, these facts argued for the conclusion that the ChMAP-28 cytotoxic effect was most likely mediated by necrotic rather than apoptotic mechanism.

In this study, we took melittin as the control cytotoxic peptide taking into account its similarity to ChMAP-28. Both peptides have alpha-helical structures. Higher selectivity of ChMAP-28 in comparison with melittin may be due to its lower hydrophobicity and higher cationicity. Calculated index GRAVY (grand average of hydropathicity) for ChMAP-28 is of -0.659, as for melittin it is of 0.273. ChMAP-28 primary structure includes eleven basic residues (Arg, Lys, His).

As for melittin-linked cytotoxicity, apoptosis and necrosis have been previously reported as alternative mechanisms of the peptide anticancer activity (Gajski and Garaj-Vrhovac, 2013). Melittin was incubated at concentrations of 1.0–6.0 μg/ml (0.35–2.1 μM) for 1–8 h and showed a time- and concentration-dependent inhibition of the gastric cancer cells SGC-7901 growth. Early apoptosis occurs via activation of the internal (mitochondrial) apoptotic signaling pathway by melittin (Kong et al., 2016). Melittin also induced apoptosis in the leukemic U937 cells by downregulating Akt signal pathways. The effects of melittin were reversed by an addition of Z-DEVD-FMK, the inhibitor of caspase-3 activation, while the maximum concentration of the peptide was of 3 μg/ml (1 μM) (Moon et al., 2008). In contrast, no apoptosis activation by melittin was observed in the colorectal adenocarcinoma cells HT-29 (Maher and McClean, 2008). Melittin also showed necrotic toxicity toward the AGS gastric carcinoma cell line (Mahmoodzadeh et al., 2015). Overall, melittin was recognized as an agent that causes primary necrosis in cells due to membrane pore formation (Janko et al., 2013).

More recently, conjugates and nanoparticles containing melittin were developed in attempts to increase its therapeutic interval and to provide more effective delivery (Rady et al., 2017). An evident advantage of ChMAP-28 demonstrated here such as higher anticancer specificity and lower hemolytic activity, suggest that the therapeutic potential of ChMAP-28 leaves behind many other host defense peptides including melittin and may be used for anticancer drug design.

## Conclusion

Summarizing the above, we studied the properties and mechanism of cytotoxicity of the novel goat cathelicidin peptide ChMAP-28 which showed a considerable potential as a putative anticancer agent. ChMAP-28 stands out from other host defense peptides by a selectivity and a lower hemolytic activity as compared to other α-helical and β-hairpin antimicrobial peptides. We showed here that its cytotoxicity was mediated by quick permeabilization of cell membrane with subsequent necrotic death and unlikely involved apoptosis.

## Author Contributions

AE performed all experimental work except microarray experiments and the ChMAP-28 recombinant expression, as well as drafted the manuscript. DK and TO coordinated the study and contributed to the conception of the work. PP performed heterologous expression of ChMAP-28 and purified the recombinant peptide. MS constructed the library of molecular pathways and analyzed gene expression data. AB planned gene expression experiments and wrote the paper. TO analyzed all the experimental data, revised the manuscript critically, and prepared it for publication.

## Conflict of Interest Statement

The authors declare that the research was conducted in the absence of any commercial or financial relationships that could be construed as a potential conflict of interest.

## Footnotes

1 www.atcc.org
