# Supplementary material for: Anticancer Activity of the Goat Antimicrobial Peptide ChMAP-28
Source: Front Pharmacol. 2018 Dec 21;9:1501. doi: 10.3389/fphar.2018.01501 (PMC6308165; doi:10.3389/fphar.2018.01501)
Supplement: Supplementary file 1 [file Data_Sheet_1.doc]

***Supplementary Material***

**Anticancer Аctivity of the Goat Antimicrobial Peptide ChMAP-28**

**Anna A. Emelianova^1^, Denis V. Kuzmin^1^, Pavel V. Panteleev^1^, Maxim Sorokin^2^, Anton A. Buzdin^1,3^, Tatiana V. Ovchinnikova^1,3,*^**

^1^M.M.Shemyakin & Yu.A.Ovchinnikov Institute of Bioorganic Chemistry, the [Russian Academy of Sciences](http://www.ras.ru/), Mikhluho-Maklaya str., 16/10, Moscow, 117997, Russia

^2^ Department of Bioinformatics and Molecular Networks, OmicsWay Corporation, Walnut, CA 91789, USA

^3^I.M. Sechenov First Moscow State Medical University, Trubetskaya str, 8/2, Moscow, 119991, Russia

*** Correspondence:**

Tatiana V. Ovchinnikova

[ovch@ibch.ru](mailto:ovch@ibch.ru)

**Supplementary Figure S1.** (A) Reversed-phase high-performance liquid chromatography (RP-HPLC) of the recombinant ChMAP-28. RP-HPLC was performed with a linear gradient from 5 to 80% (v/v) of acetonitrile in water containing 0.1% TFA within 1 h. The fraction of the mature recombinant peptide is marked with an asterisk. (B) MALDI-MS analysis of the recombinant ChMAP-28 peptide. The experimental [M+H]^+^ monoisotopic *m/z* is presented in the picture.

**Supplementary Table S1.** Pathway activation strength (PAS) and case-to-normal (CNR) values for unaffected and ChMAP-28-treated cells (at the peptide concentration of 1.25, 2.5, and 5 µM).

**Supplementary Figure S2**. Schematic representation of alterations in Notch and GSK3 molecular pathways after 6 h of incubation with ChMAP-28 at the peptide concentration of 1.25, 2.5, and 5 µM. The pathway is shown as an interacting network, where green arrows indicate activation, red arrows – inhibition. PAS is shown for each sample. Color depth corresponds to the logarithms of the CNR expression rate for each node, where “normal” is a geometric average between control samples. Exact CNR values are provided in Supplementary Table S1

**Supplementary Figure S3**. Schematic representation of alterations in caspase cascade pathway after 6 h of incubation with ChMAP-28 at the peptide concentration of 1.25, 2.5, and 5 µM. The pathway is shown as an interacting network, where green arrows indicate activation, red arrows – inhibition. PAS is shown for each sample. Color depth corresponds to the logarithms of the CNR expression rate for each node, where “normal” is a geometric average between control samples. Exact CNR values are provided in Supplementary Table S1
