# Supplementary figures and images for: Anticancer Activity of the Goat Antimicrobial Peptide ChMAP-28
Source: Front Pharmacol. 2018 Dec 21;9:1501. doi: 10.3389/fphar.2018.01501 (PMC6308165; doi:10.3389/fphar.2018.01501)

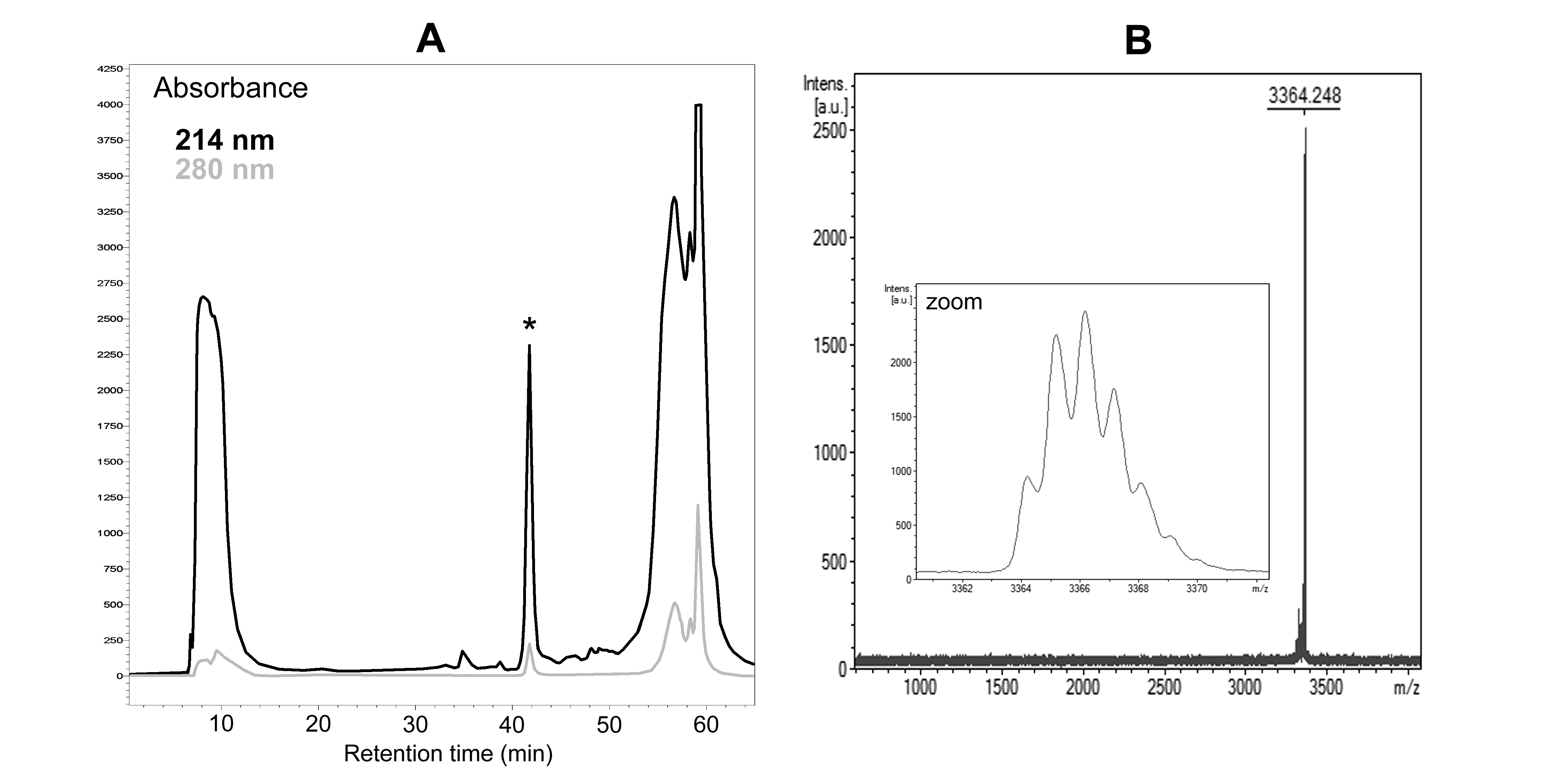

Supplement: Supplementary file 2 [file Image_1.tif]

Notch\_Signaling\_Pathway

GSK3\_Signaling\_Pathway

ChMAP-28

1.25 μM

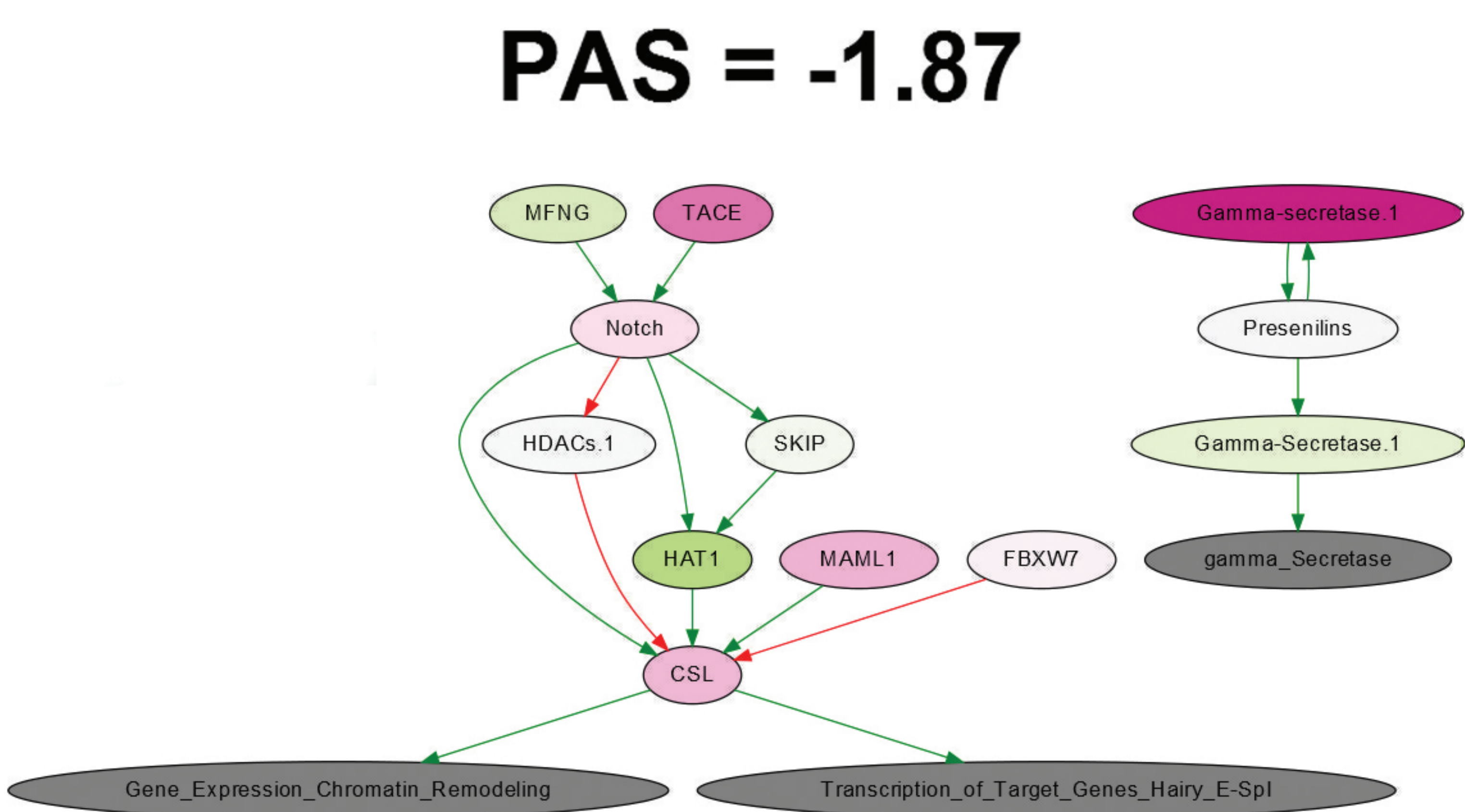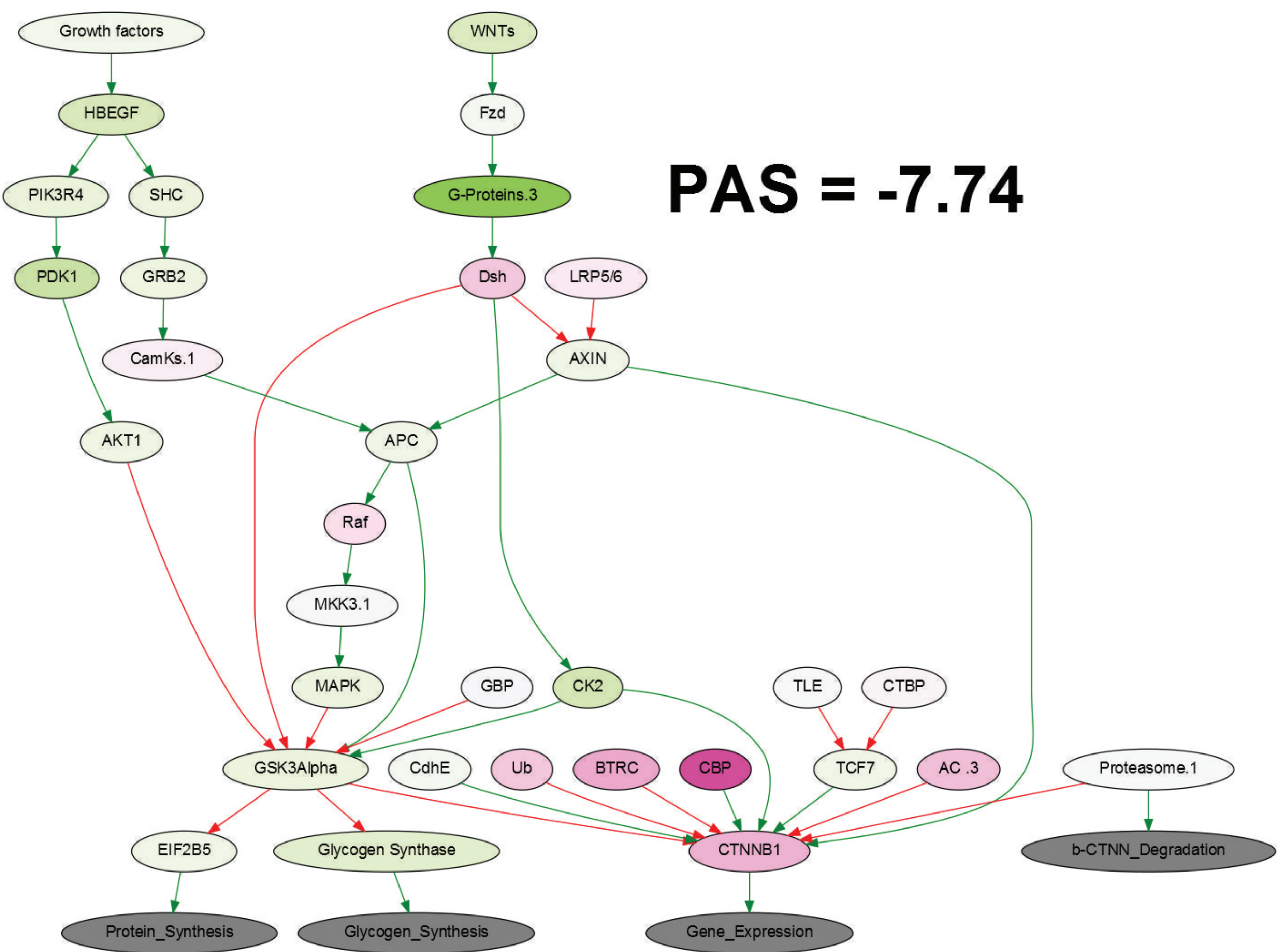

2.5 μM

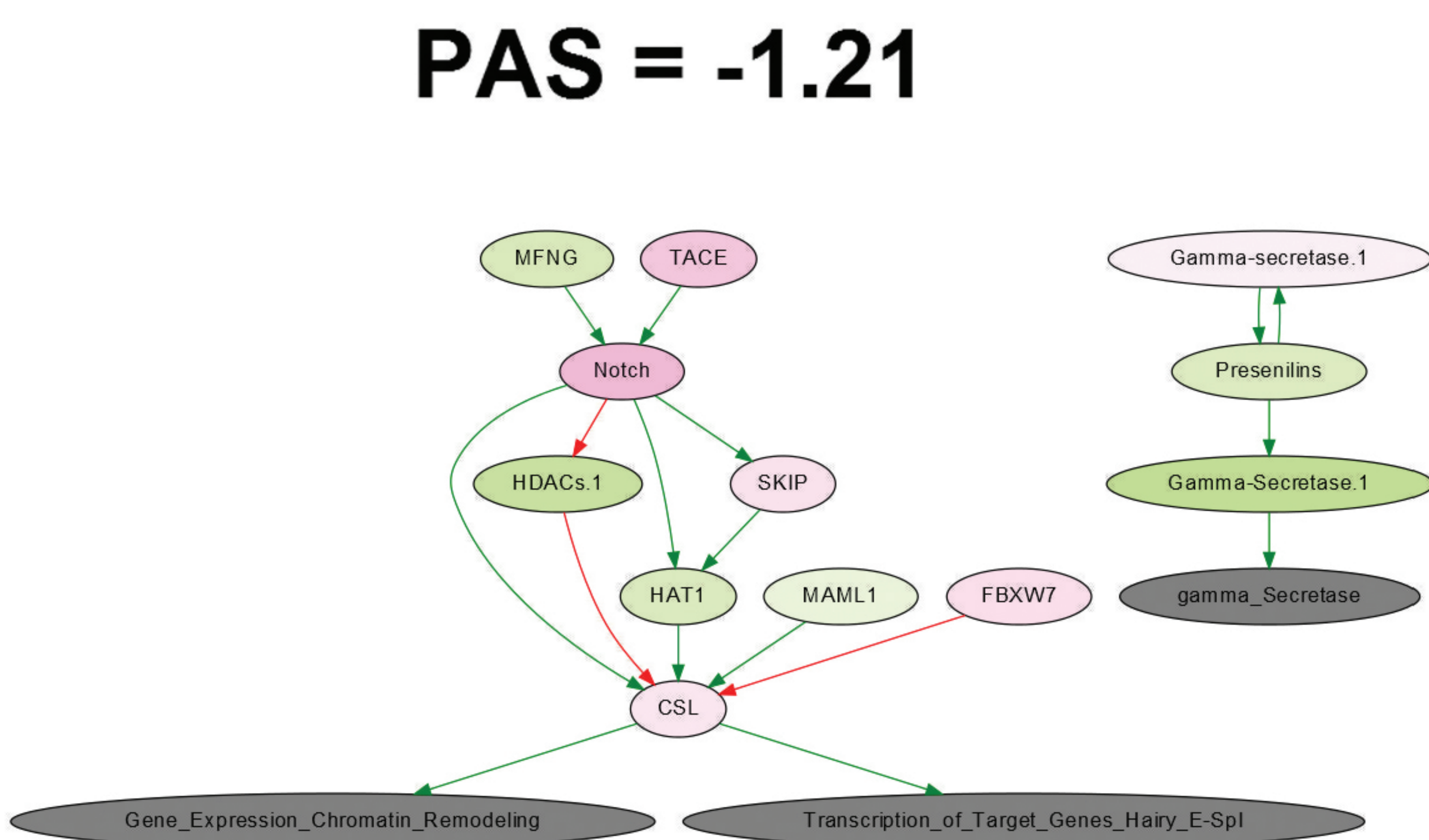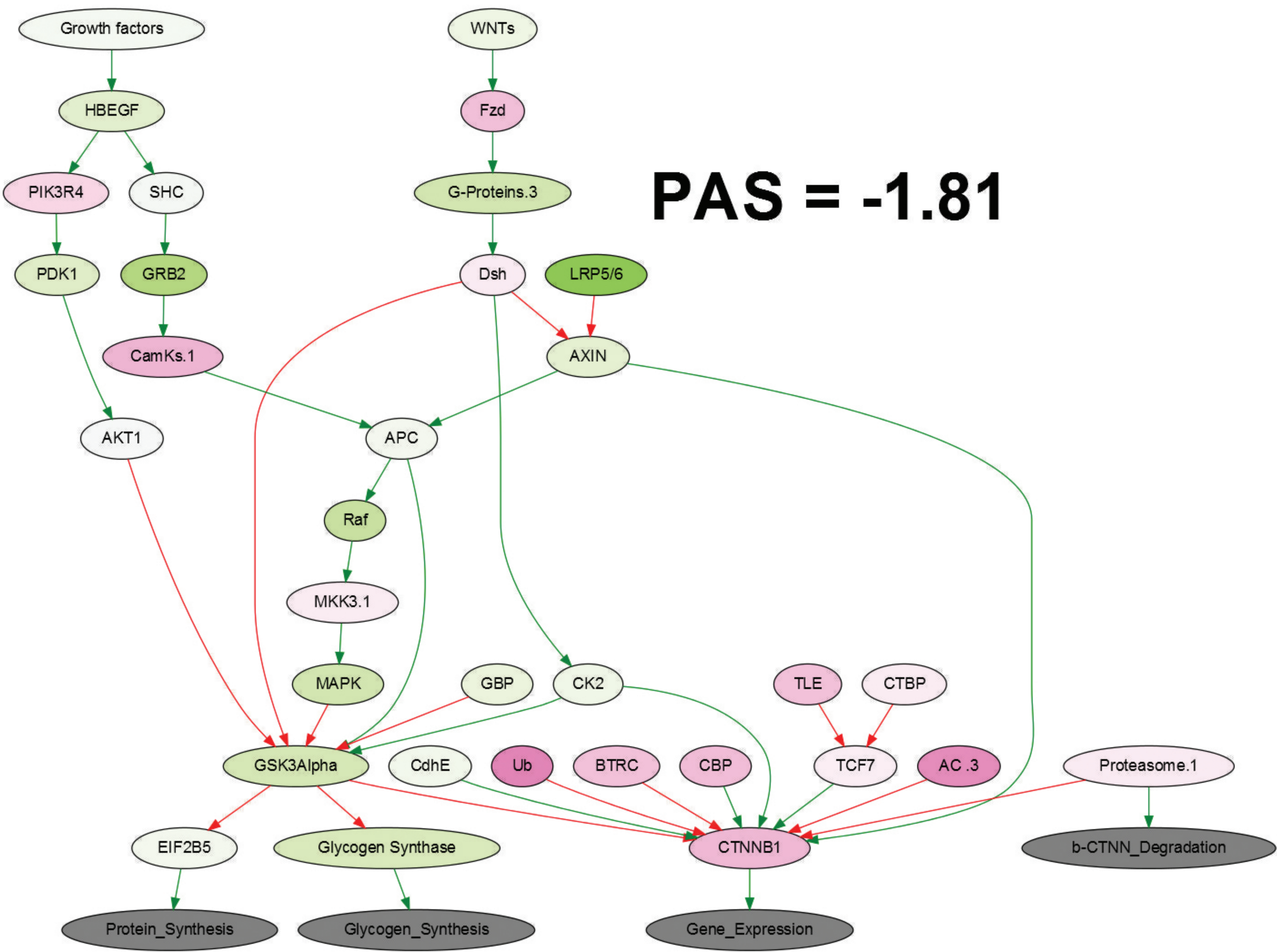

5 μM

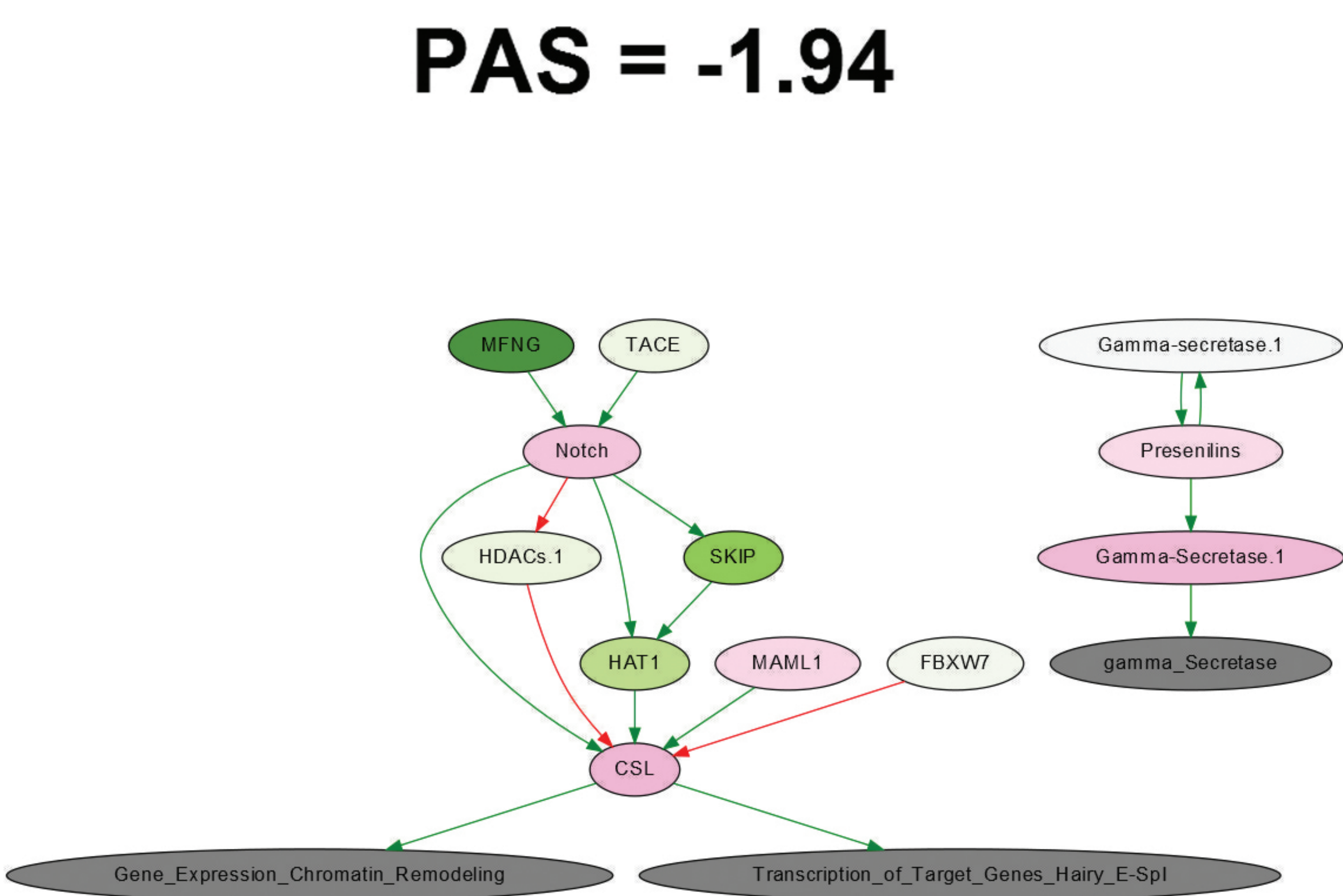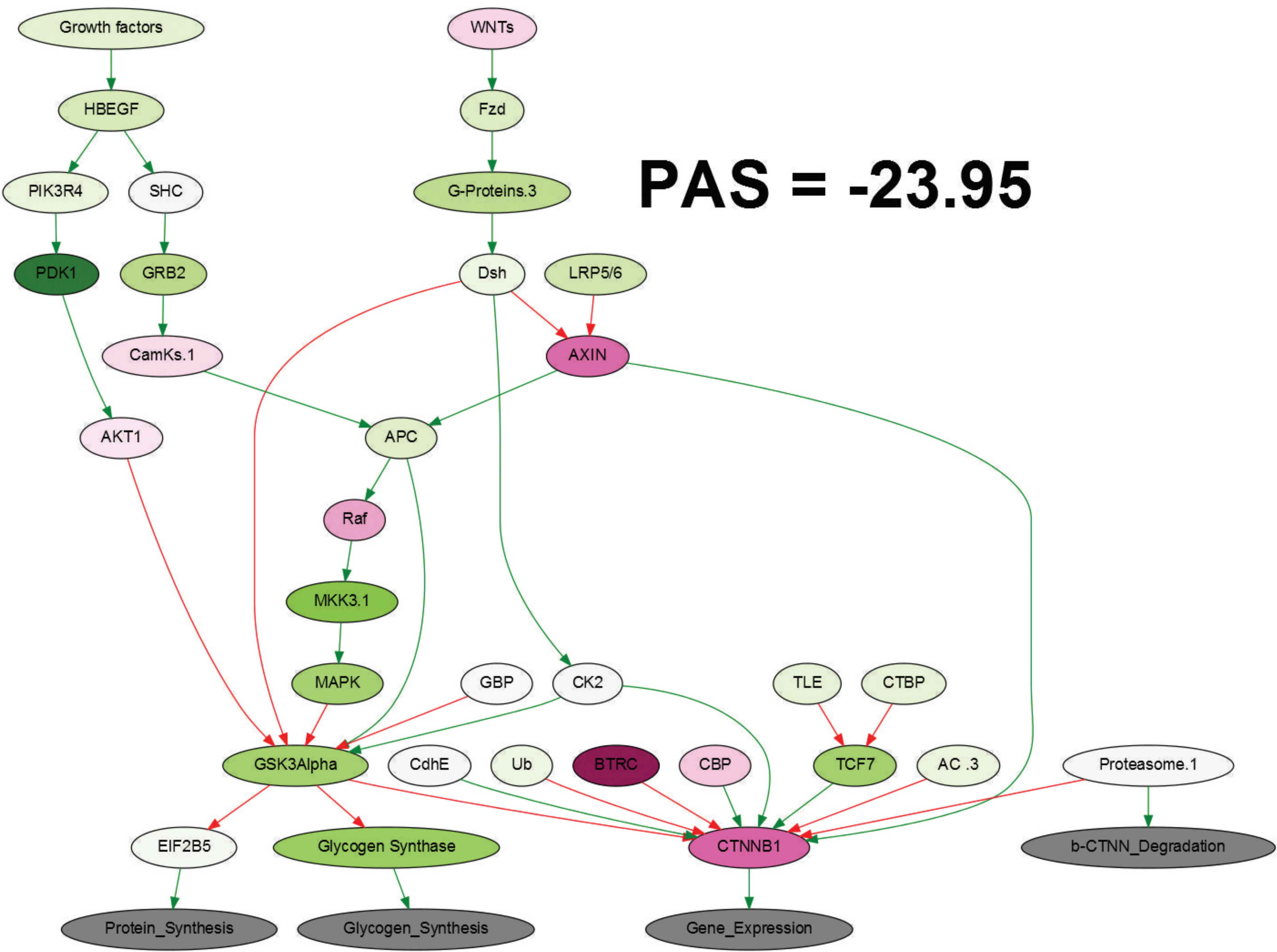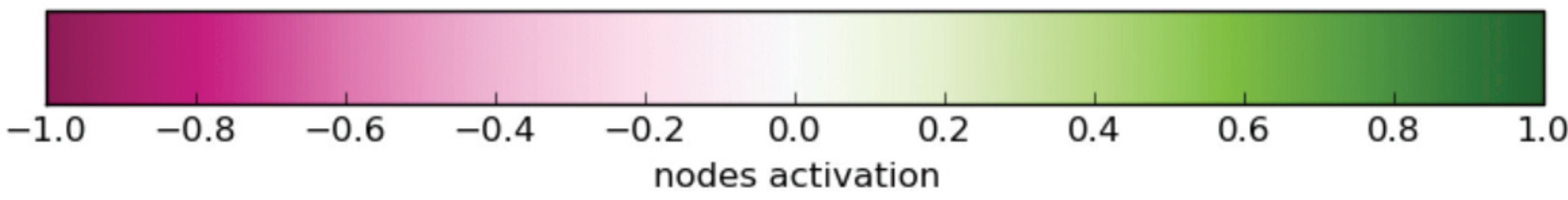

Supplement: Supplementary file 3 [file Image_2.pdf]

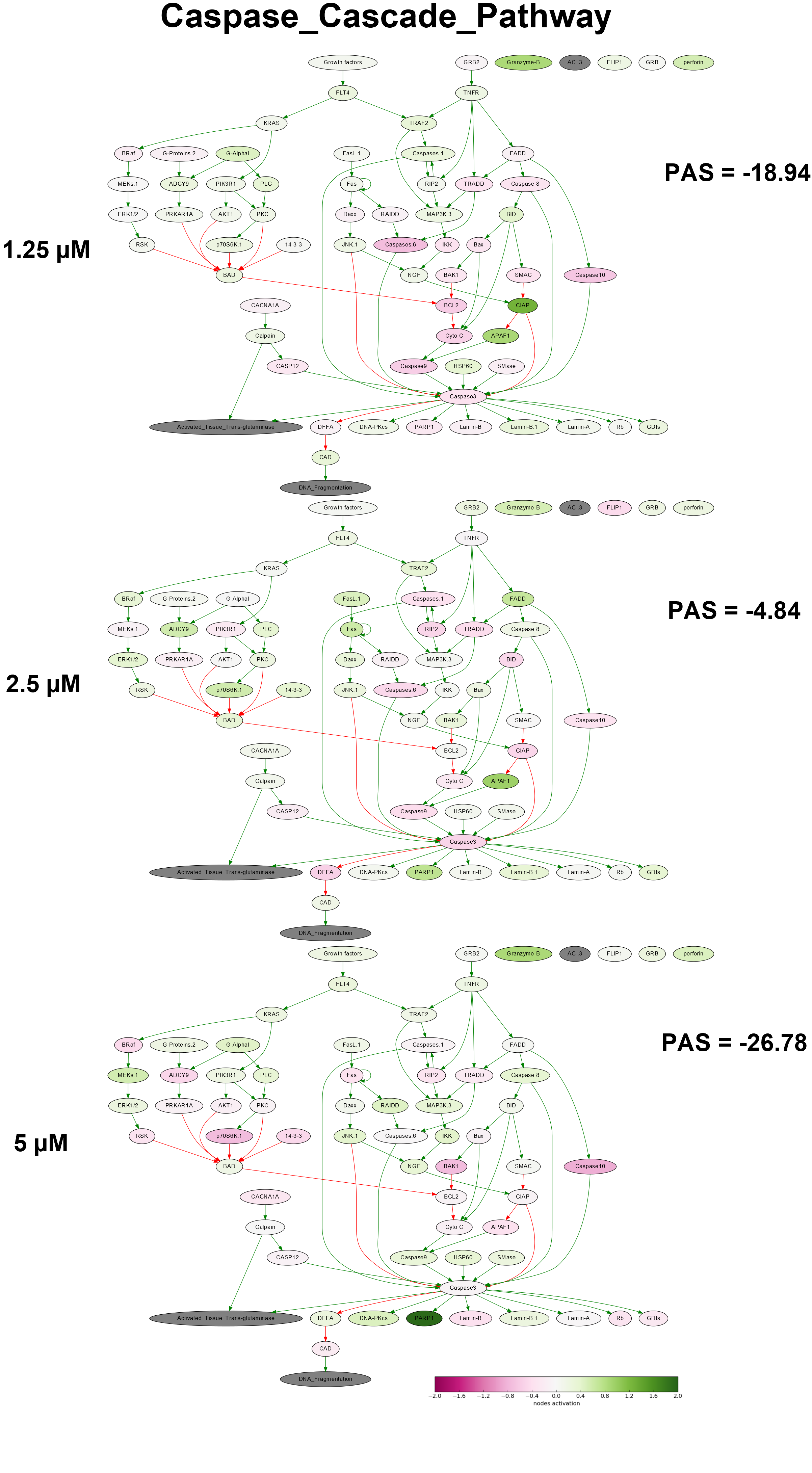

Supplement: Supplementary file 4 [file Image_3.png]
